# Investigating the Contribution of the Imprinted Gene *Asb4* to Parental Care

**DOI:** 10.1111/ejn.70655

**Published:** 2026-09-07

**Authors:** Rachel‐Ann Jones, Matthew J. Higgs, Rosalind M. John, Anthony R. Isles

**Affiliations:** ^1^ Centre for Neuropsychiatric Genetics and Genomics, Neuroscience and Mental Health Innovation Institute, School of Medicine Cardiff University Cardiff UK; ^2^ School of Biosciences Cardiff University Cardiff UK

**Keywords:** *Asb4*, epigenetics, genomic imprinting, imprinted genes, medial preoptic area, neuronal circuits, parental care behaviour

## Abstract

Genomic imprinting is a form of epigenetic regulation that leads to expression from one parental allele only and, in animals, is unique to mammals. Imprinted gene expression is predominant in the brain and their roles in neural processes are becoming more appreciated. Recent analyses have indicated an enrichment of imprinted gene expression in the ‘parental hub’ circuitry. Specifically, imprinted genes were over‐represented in the transcriptomic profile of galanin positive (Gal^+^) neurons in the medial preoptic area (MPOA) of the hypothalamus. One of those imprinted genes showing enriched expression in Gal^+^ neurons was the maternally expressed *Asb4*. Here, we propose that *Asb4* supports normal activation of MPOA Gal^+^ neurons required for parental care. We aim to demonstrate abnormal parental behaviours—such as decreased pup retrieval and impaired nest building—and activity of MPOA Gal^+^ neurons in brain‐specific maternal *Asb4* knockout mice and hypothesis knockout animals will demonstrate a deficient behavioural phenotype in one or more parental behavioural characteristics.

## Graphical abstract

Genomic imprinting is enriched in neural circuits regulating parental behaviour, such as medial preoptic area (MPOA). We hypothesize that maternal *Asb4* promotes activation of MPOA Gal^+^ neurons required for normal parental care. Using brain‐specific maternal *Asb4* knockout mice, we will assess parental behaviours and neuronal activation. We predict that loss of maternal *Asb4* will reduce MPOA Gal^+^ neuron activity, resulting in impaired pup retrieval, nest building and caregiving behaviours.

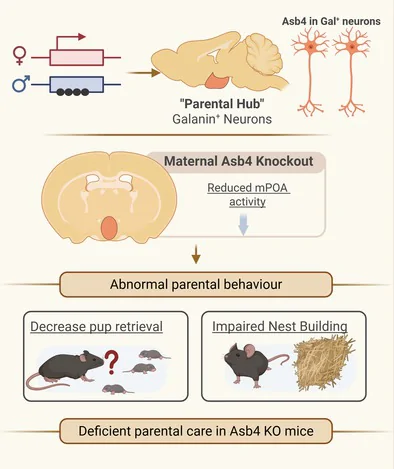

**Abbreviations**
ANOVA: analysis of variance
*Asb4*
: Ankyrin repeat and SOCS box containing 4
BORIS: Behavioural Observation Research Interactive Software
Brs3: bombesin receptor subtype‐3
Calcr: calcitonin receptor
CI: confidence interval
Gal: galanin
GLMMs: generalized linear mixed models
GLMs: generalized linear models
IG: imprinted gene
KO: knockout
MPOA: medial preoptic area
PCA: principle component analysis
Peg1: paternally expressed gene 1
Peg3: paternally expressed gene 3
ROI: region of interest
scRNA‐seq: single cell RNA sequencing
SESOI: smallest effect size of interest
Syn1: synapsin I
Th: tyrosine hydroxylase
TOST: two one‐sided tests procedure
WT: wild type

## 1 Introduction

Genomic imprinting is an epigenetic phenomenon found in mammals and flowering plants in which gene expression is determined by whether an allele is maternally or paternally inherited (Scott and Spielman 2006). In mammals, imprinted gene (IG) expression plays a critical role in both embryonic development and adult tissue function (Bartolomei and Ferguson‐Smith 2011). The brain has consistently been shown to be an organ in which a large number of IGs are expressed. Indeed, IGs are over‐represented in the adult mouse brain compared with other adult tissues, and IG expression is often highly specific to brain region and cell type (Tucci et al. 2019; Higgs et al. 2022). In particular, multiple studies have described a higher frequency of IG expression in the hypothalamus over any other brain region (Gregg et al. 2010; Ivanova and Kelsey 2011; Babak et al. 2015; Higgs et al. 2022). The hypothalamus is a key regulator of many endocrine functions and is involved in the regulation of energy balance through its influence on food intake, metabolic rate and body temperature. Several key hypothalamic‐related behaviours, such as feeding, sleep and parental caregiving, have been repeatedly linked to IGs (Bischof et al. 2007; Kozlov et al. 2007; Lassi et al. 2012; Higgs et al. 2023).

We have taken advantage of existing single cell (sc)RNA‐seq data to examine IG expression in the defined ‘parental hub’ circuitry of the hypothalamus. Recent work has identified the specific neuron‐types within this circuitry, showing a critical role for galanin (Gal) neurons co‐expressing tyrosine hydroxylase (Th), calcitonin receptor (Calcr) or bombesin receptor subtype‐3 (Brs3) within the medial preoptic area (MPOA) of the hypothalamus as the hub neurons required to produce the specific facets of parenting behaviour in mothers, fathers and virgins. We demonstrated that IGs as a group were over‐represented in the transcriptomic profile of Gal^+^ neurons in the MPOA and that this expression had functional relevance for parental care (Higgs et al. 2023). Of the 33 IGs identified, this study also highlighted Ankyrin repeat and SOCS box containing protein 4 (*Asb4*) as a maternally expressed enriched IG in the Gal^+^ neurons of the MPOA of the hypothalamus. Consequently, *Asb4* may mediate a wide range of behaviours associated with parenting in both males and females. This is exciting, as most studies caregiving behaviour in the context of genomic imprinting have focused on paternally expressed IGs such as Peg1 (Lefebvre et al. 1998), Peg3 (Li et al. 1999) and Magel2 (Higgs et al. 2023). *Asb4* has previously been shown to influence food intake, somatic growth and metabolic rate (Li et al. 2005; Li et al. 2010; Vagena et al. 2022). However, the function of *Asb4* within the hypothalamus remains to be fully understood. We predict that *Asb4* function in the MPOA of the hypothalamus is required for parental behaviour.

### 1.1 Hypothesis

In this study, we aim to fully characterize parental behaviour in a neuron specific *Asb4* knockout (KO) mice and hypothesize that deletion of maternal *Asb4* in Gal^+^ neurons will result in a decline of parental behaviour in primiparous mothers (H1) and in primiparous fathers (H2). Virgin females can display ‘spontaneous maternal behaviour’ in which a certain proportion will display full maternal behaviour towards pups when first exposed. Therefore, we hypothesize that virgin female KO mice will display fewer maternal tendencies compared with wild‐type (WT) virgin mice (H3), as summarized in Table 1.

**TABLE 1:** Research project summary table.

Research question	Hypothesis	Sampling plan	Statistical analysis	Outcome
Does *Asb4* KO in the MPOA effect parental caregiving behaviours in primiparous mothers?	We predict KO mothers to display a deficit in some or all care‐giving characteristics (H1)	32 primiparous mothers, primiparous fathers or virgin females will be tested on their ability to carry out caregiving activities by being subjected to a combined nest building and pup retrieval test, and a three chambers pup preference test. The following metrics will be recorded to determine the effect of *Abs4* KO on caregiving behaviours: Total time taken to complete task (Ha) Nest quality will be judged on a 1–5 scale as described by Deacon (2006) (Hb) Pup preference score (Hc) The number and proportion of total time spent carrying out qualitative parental behaviour characteristics listed in Table 3 (H1d) and motility and velocity analysis (H1e) will be recorded and used as supplementary metrics of parental behaviour.	H1/2a, H1/2c and H1/2e: Analysed using one‐way ANOVA with Tukey's post hoc test, Cox proportional hazards models or Kruskal–Wallis test with Holm–Bonferroni‐corrected Dunn's post hoc test, depending on whether assumptions are met. H1/2b: Analysed using the Kruskal–Wallis test with Holm–Bonferroni‐corrected Dunn's post hoc test. H1/2d: Analysed using Chi‐squared test or Fisher's exact test, and generalized linear models.	Hypothesis will be supported if *Asb4* KO mothers exhibit a statistically significant change (*p* < 0.05) AND at least medium effect size (β ≥ 0.3, *r* ≥ 0.3) For outcomes with *p* ≥ 0.05, equivalence testing using the TOST procedure with predefined SESOI bounds will be performed to assess whether effects are small enough to support the null hypothesis.
Does *Asb4* KO in the MPOA effect parental caregiving behaviours in primiparous fathers?	We predict KO fathers to display a deficit in some or all care‐giving characteristics (H2)			
Does *Asb4* KO effect parental caregiving behaviours in virgin females?	We predict KO virgins to display a deficit in some or all care‐giving characteristics (H3)		H3a, H3c and H3e: Analysed using two‐way mixed ANOVAs with genotype and exposure as variables and Holm–Bonferroni corrected *t*‐tests or generalized linear mixed models depending on whether assumptions are met. H3b and H3d analysed by Friedman test and Holm–Bonferroni‐corrected Dunn tests	Hypothesis will be supported if *Asb4* KO mothers exhibit a statistically significant change (*p* < 0.05) AND at least medium effect size (β ≥ 0.3, *r* ≥ 0.3) For outcomes with *p* ≥ 0.05, equivalence testing using the TOST procedure with predefined SESOI bounds will be performed to assess whether effects are small enough to support the null hypothesis.
Does *Asb4* KO in the MPOA impact neuronal activity in the POA?	We predict KO will reduce the activity of Gal^+^ neurons (H4)	24 animals per genotype (six animals per group) will be used to examine the effect of *Asb4* KO on c‐Fos expression in Gal^+^ cells in the MPOA. This will be assessed using RNAscope. Mice will be pup‐exposed to activate neurons. The control group will be made up of mice not exposed to pups. The number of c‐Fos molecules per Gal^+^ neuron will be normalized to counts per 1000 POA cells to account for variability in sections and POA position per animal.	Results will be compared using three‐way ANOVA, with genotype, exposure and sex as fixed factors and post hoc Holm–Bonferroni correction pairwise *t*‐tests.	Hypothesis (H4) will be supported if KO mice show a significantly different normalized c‐Fos count following pup exposure compared with WT mice (*p* < 0.05) AND at least medium effect size (β ≥ 0.3, *r* ≥ 0.3) For outcomes with *p* ≥ 0.05, equivalence testing using the TOST procedure with predefined SESOI bounds will be performed to assess whether effects are small enough to support the null hypothesis.

## 2 Experimental Procedures

### 2.1 Mice

All procedures will be conducted in accordance with ethical guidelines and under United Kingdom Home Office project licence PP1850831 (Anthony R. Isles). All mice will be housed under standard conditions with access to enrichment (cardboard fun‐tunnel and nesting material) throughout the study on a 12 h light–dark cycle with lights coming on at 0800 h, a temperature range of 21°C ± 2°C and with free access to tap water and standard chow.

*Asb4* ^flox/flox^ mice (Vagena et al. 2022) will be rederived in Cardiff University from cryopreserved sperm samples provided by Prof Allison W. Xu at University College San Francisco (MTA with Cardiff University in place). *Asb4* is expressed in many organ systems, and it is known that *Asb4* KO in the placenta causes a pathology that phenocopies human pre‐eclampsia (Townley‐Tilson et al. 2014). To limit the effects to the brain, we propose the use of Syn1‐cre mice (B6.Cg‐Tg(Syn1‐cre)671Jxm/J) to restrict KO of *Asb4* to neurons. Neuron‐targeted *Asb4* KO mice will be generated using Cre‐LoxP system. The Syn1‐cre mice will be obtained from The Jackson Laboratory, USA (JAX stock #003966). The animals used in the study will come from the F1 generation. Syn1‐Cre line will be mated with an *Asb4* ^flox/flox^ animal to produce *Asb4* ^flox/+^/Syn1‐Cre experimental animals.

### 2.2 PCR Genotyping

Animals will be genotyped via PCR using the following primer pairs CSD‐Asb4‐F TCAGCTCTCTGCTCTTTCTTCCAGG and CSD‐Asb4‐ttR. CTCTTACAGTCTCTACGGCTCCTCG producing two bands 516 bp (WT allele) and 674 bp (Pre‐Cre allele). CSD‐Asb4‐F TCAGCTCTCTGCTCTTTCTTCCAGG and CSD‐Asb4‐R AGCAGCTCATTCATGAAAAGGGACG producing a single band at 592 bp indicating deletion. Cre animals will be detected by following primers: forward 15663 CTCAGCGCTGCCTCAGTCT, reverse 15664 GCATCGACCGGTAATGCA producing a single band at 300 bp for the Syn1‐Cre transgene. PCR will be carried out using the following reaction mix to a final volume of 20 μL: 1.35 mM PCR buffer (Promega), 0.4 M Betaine, 1.5 mM MgCl, 200 μM dNTPs, 0.2 μM each primer, 1 μL DNA, 5 U Taq DNA Polymerase (Promega). PCR will be performed under the following thermocycling conditions: Initial denaturation, 95°C for 10 min. 34 cycles of 95°C for 30 s, 65°C for 30 s, 72°C for 1 min. Final extension step 72°C for 5 min.

### 2.3 DNA Extraction

Lysis buffer (0.1 M Tris–HCl pH 8.5, 5 mM EDTA pH 8.0, 0.02% SDS, 0.2 M NaCl, 1% [v/v] proteinase K) will be added to 10–30 mg tissue of tissue sample and incubated at 55°C on a heating block overnight. Samples will be centrifuged at 13,000 rpm for 10 min and supernatant transferred to fresh sterile Eppendorf. 1:1 volume of cold phenol will be added to each sample and mixed rigorously for 10 min. Then centrifuged at 13,000 rpm for 10 min. The upper aqueous phase transferred to a fresh sterile Eppendorf. 1:1 volume of ice‐cold isopropanol will be added to each sample and mixed thoroughly to precipitate the DNA. DNA will be pelleted by centrifuging samples at 13,000 rpm for 10 min at 4°C. The supernatant is discarded, and the DNA pellets are resuspended in 80% ethanol and centrifuged at 13,000 rpm for 5 min. Again, the supernatant discarded. The DNA pellet will be dried at 65°C for 10 min and resuspended in 50 μL of TE buffer (10 mM Tris pH 8.0, 1 mM EDTA pH 8.0) and stored at −20°C until required.

### 2.4 KO Validation

KO will initially be established by PCR genotyping of adult brain, liver and placenta. This is to ensure deletion is restricted to neurons as per Syn1‐Cre. Placenta from E16.5 will also be tested to ensure specificity of KO and no compounding effects on maternal behaviour given the known placental role of *Asb4* (Townley‐Tilson et al. 2014). We will then examine brains, via RNAscope, for *Asb4* and *Galanin* expression, to ensure *Asb4* KO is successful in our neurons of interest. In the *Asb4* ^flox/flox^ mice the LoxP sites flank exon 3. This floxed exon results in the production of a loss‐of‐function truncated protein lacking the ankyrin repeats of *Asb4*. Therefore, we shall use an *Asb4* RNAscope probe designed to recognize exon 3. Successful KO of maternal *Asb4* will be classified as Gal expressing cells that contain *Asb4* detection equal to or below that of no probe control in wild type animals.

We will validate successful deletion using three mice from each of the following groups: Wild type (WT; true wild type, basal control), *Asb4* ^flox/+^ (functionally WT), Syn1‐cre (functionally WT) and neuron targeted maternal *Asb4* KO (*Asb4* ^flox/+^/Syn1‐cre; experimental group) will be assessed. An additional group, *Asb4* ^+/flox^/Syn1‐cre, in which the *Asb4* ^flox^ allele is paternally inherited, will also be probed. This group should genotype as KO individuals, but *Asb4* expression should be detectable as normal via RNAscope. This analysis will confirm that *Asb4* is imprinted in Gal^+^ neurons. We expect to see *Asb4* present in Gal^+^ neurons in the WT and paternal KO groups but not the maternal KO groups. As this is a binary question, we anticipate three mice per group to be sufficient power to establish if KO was successful.

### 2.5 Behavioural Methods

**FIGURE 1:**
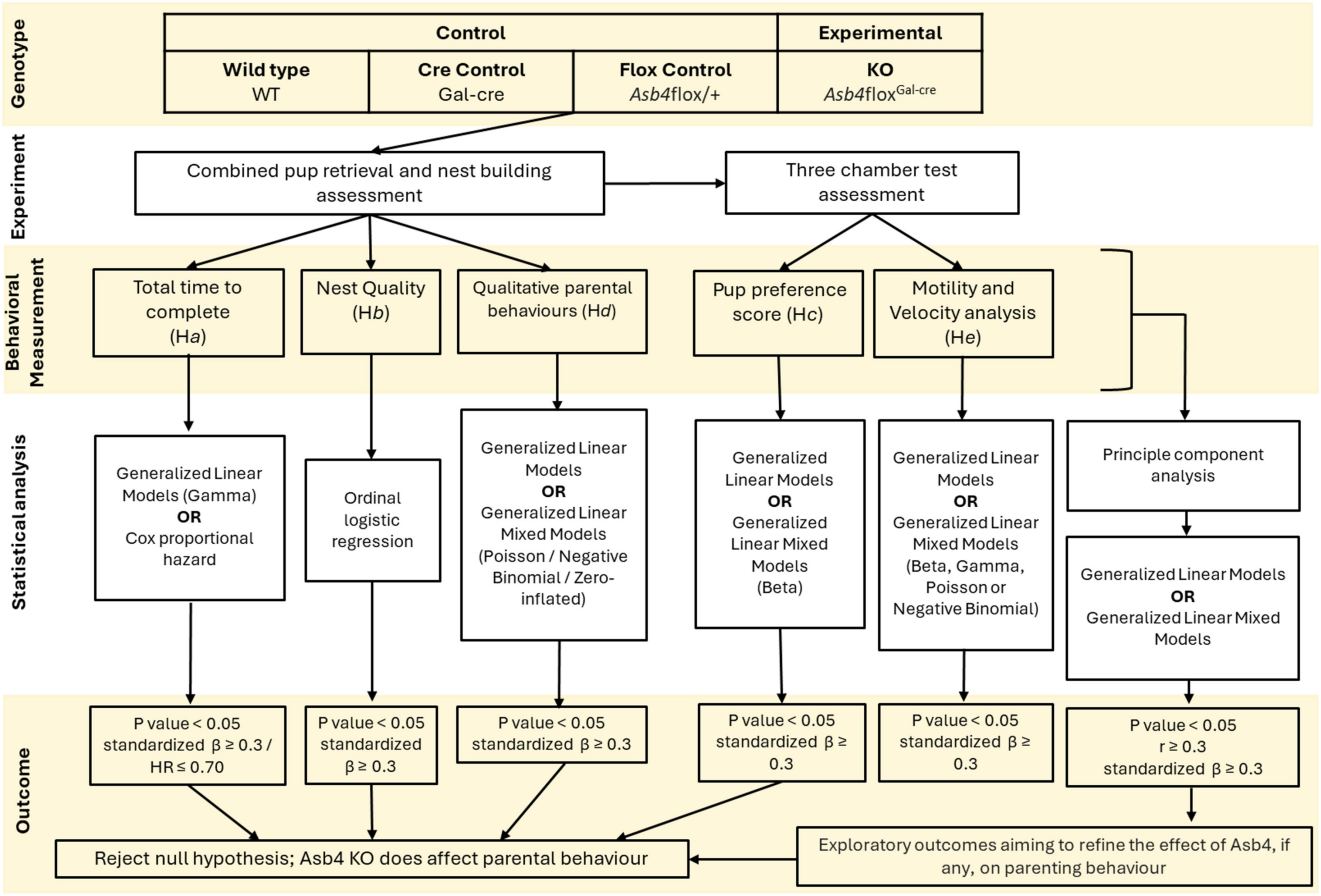
Overview of behavioural analysis pipeline for mothers and fathers. Schematic showing the experimental design, behavioural assessments, statistical analyses and predefined criteria used to evaluate the effect of *Asb4* deletion on parental behaviour in mothers and fathers.

**FIGURE 2:**
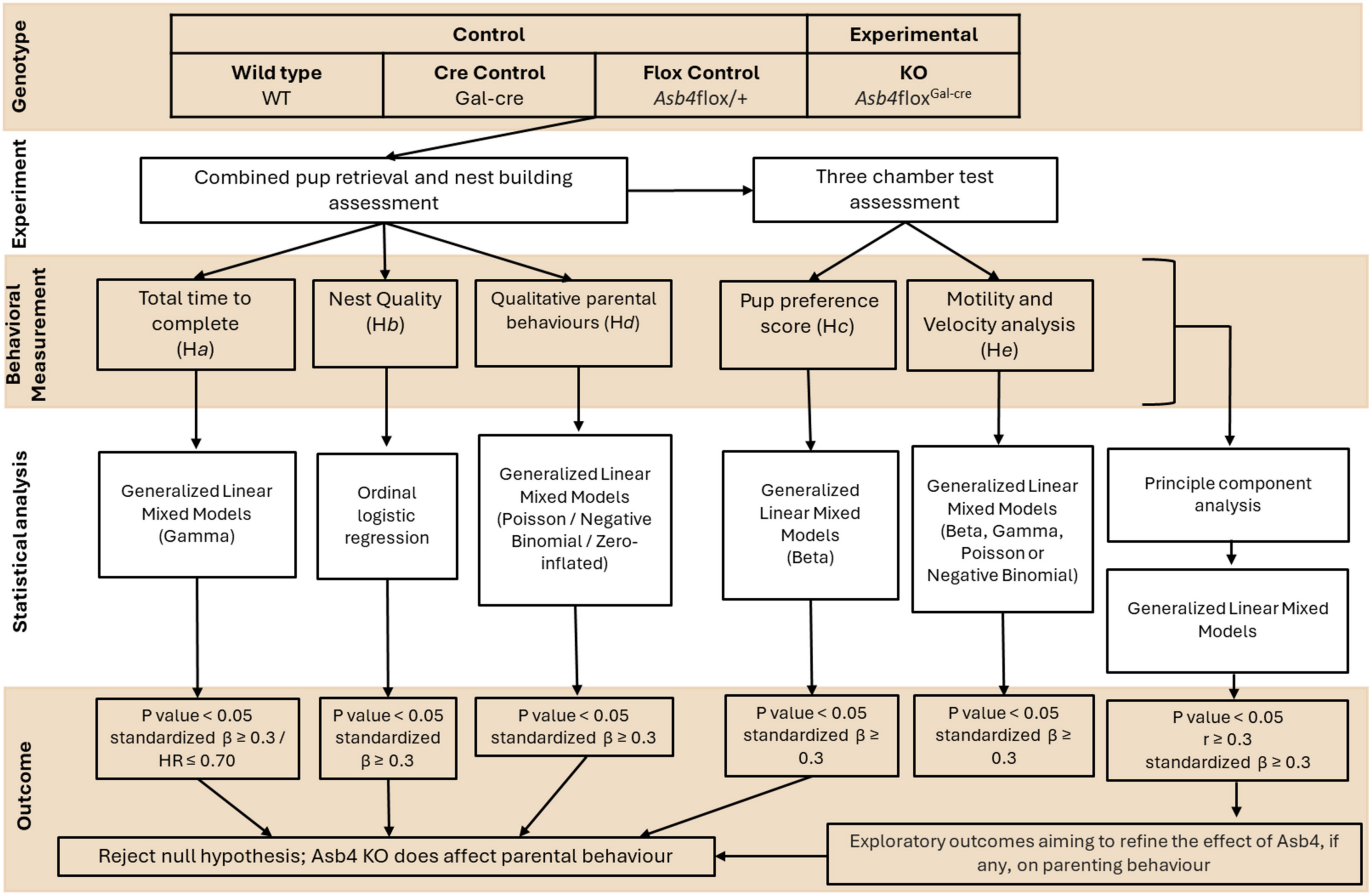
Overview of behavioural analysis pipeline for virgin females. Schematic showing the experimental design, behavioural assessments, statistical analyses and predefined criteria used to evaluate the effect of *Asb4* deletion on parental behaviour in mothers and fathers.

All mice will be tested using with the same three pups for their assessment. For mothers and fathers, this will be three pups from their own litters. For virgin females, litters will be produced from separate WT × WT crosses and each set of three pups will be used with only one virgin female. All behavioural tests will be carried out during the light phase of the light cycle (between 08:00–20:00) in a dimly lit room (< 30 lx). All mice will be handled using the tunnel technique to avoid undue stress before and after the tests. All mice will carry out a retrieval/nest building assessment followed by a three chambers assessment. Following habituation, fathers will perform the retrieval assessment when test pups are P3, mothers will perform the retrieval assessment when test pups are P4. Both will carry out the three chambers test on P5. Virgin females will perform the retrieval assessment when test pups were P3 and P4 and also carry out the three chambers assessment on P5. All behavioural tests will be conducted blind to genotype. An overview of behavioural analysis pipeline can be found in Figure 1 for mothers and fathers, and Figure 2 for virgin females.

### 2.6 Retrieval and Nest Building Assessment

Animals will be tested within their home cage, which will be cleaned the day after the litter was born (2 days prior to testing) by replacing soiled bedding and sawdust. For the test, the home cage will have all enrichment removed and placed, with metal cage lid removed, inside a plastic storage container. All mice will be given the same amount of nesting material before their litter is born. Directly prior to the test, the test animal (mother/father/virgin female) will be removed from the home cage and placed in a holding cage. The other animal in the pair will be placed in a new clean cage with the enrichment from the home cage, a fresh nest disc and all the pups except the three pups used for testing. The three test pups will remain in the home cage and positioned against the short ends of the cage, opposite the home nest with two in the corners and one directly in between these two. The home nest will be shredded completely and placed all the way along the opposite side of the cage from where the pups were placed. Video recording will be started then test animals will be returned to their home cage, placed directly onto the shredded nest. Animals will have one‐hour in which to complete the behaviour test. The goal of which is to retrieve the three scattered pups to the nest material and to re‐construct the home nest from the scattered material.

### 2.7 Mothers and Fathers

Mice will be paired together at aged 9–12 weeks; females will be weighed periodically to confirm pregnancies, and litters will be born to mice aged 12–17 weeks. On Day 1 postpartum (P1), the home cage (with the mother, father and pups) will be carried to the test room, and on Day 2 postpartum (P2), the cage lid will be removed to allow for habituation. Directly prior to the tests on P3 (father)/P4 (mother), the animals (dam, sire, pups) will undergo a 20‐min habituation period following the removal of all enrichment items from the cage. All non‐test animals, including any remaining pups, will be removed from the test room before testing begins. Biological offspring will be used for all experiments, with the exception of virgin females. Based on Mendelian inheritance, approximately 25% of pups are expected to be maternal *Asb4* ^−/+^‐Syn1‐cre KOs. There is currently no data on early‐life abnormalities (e.g., growth deficits, motor delays, altered maternal care) in the *Asb4* ^−/+^‐Syn1‐cre line, and this is difficult to predict. Pup outcomes (e.g., weight, survival) will be monitored during initial characterization of the line and throughout the study to identify any emergent phenotypes. All pups used in behavioural assays will be genotyped, and the data will be retrospectively re‐analysed excluding those tests involving KO pups. Additionally, we will report any observed differences, such as whether adults retrieve KO pups later or show a preference for litters without KO pups.

### 2.8 Virgin Females

Virgin female mice will be housed in same genotype pairs. Once three pups have been assigned to a virgin female, they will be habituated to the test environment on day P1 and P2 (identical to the mothers and fathers). The virgin females will then be tested twice, once on P3 and once on P4. The pair of females in a cage will be tested one after another; the order of testing will be reversed on the second day. Females exposed to pups will not be reintroduced to females that have not been exposed until their test was also carried out. All pups for virgin female assessments will be derived from WT × WT crosses to ensure all pups are WT.

### 2.9 Three Chambers Pup Preference Assessment

On P5, all animals will carry out a three chambers assessment with the same three pups as used in the retrieval test. The three chambers apparatus will consist of a white Perspex arena (40 × 30 × 30 cm, h × w × d) divided into three equal chambers connected in a row. Two guillotine doors (5 × 5cm, operated by a pulley system) will be used to connect each of the exterior chambers to the middle chamber. For the pup preference assessment, mice will be habituated to the middle chambers for 5 min with the guillotine doors closed. After this, three pups (and fresh bedding) will be placed at the outer edge of one of the exterior chambers with a protective cage placed over the top of the pups and weighted down. At the outer edge of the other exterior chamber, a novel object (a large Lego brick and an equivalent amount of fresh bedding) will be placed and covered with an identical protective cage. The two guillotine doors will then be opened simultaneously. From this point, the test mouse has 10 min in which to freely explore all chambers. Apparatus will be thoroughly wiped down between trials and the chamber containing pups will be alternated between trials. Pup preference will be calculated as both (1) the difference in time spent with pups vs. novel object and (2) the ratio of pup interaction time to total stimulus investigation in order to reflect both absolute pup preference and control for total exploration of each individual. Total investigation time (seconds) will be treated as a continuous outcome in all primary analyses. For descriptive purposes only, animals with total investigation time < 10 s will be flagged as ‘low‐exploratory’ to contextualize behavioural variability.

### 2.10 Behaviour Metrics

To capture behaviour metrics, a high‐definition webcam will be attached to a table mounted tripod and set up positioned directly above the centre of the home cage. For the combined Retrieval and Nest Building test, the test began from the instance that the test mouse first sniffed any of the pups in the trial. From here, the time taken to retrieve each of the pups to the nest area, the time taken to construct a nest of sufficient quality and the time taken to complete the trial (retrieve all three pups to a suitable quality nest) will be recorded in milliseconds using Behavioural Observation Research Interactive Software (BORIS). The quality of the final nest built will be judged on a 1–5 scale as described by (Deacon 2006) when either the task is complete or when the one‐hour time limit had expired. In addition, the amount of time that the animals spend performing pup‐directed behaviour (sniffing pups, licking pups, grooming pups, carrying pups, nest building while pups are inside the nest, crouching/sitting in the nest while pups are inside) and self‐grooming will be recorded. Scores will be generated as a proportion of the total time it took animals to finish the task. The three chambers assessment will be scored automatically using Ethovision. The number of seconds that the test mice spent in the pup chamber compared with the object chamber and the amount of time the test animal spent within a 15‐cm diameter of the pup cage or object cage will be reordered. Motility analysis will also carried out using Ethovision. Velocity will be calculated during the retrieval and three chambers' tests as well as the number of chamber crosses that each group performed during the three chambers test.

### 2.11 Tissue Collection/Genotyping

Following behavioural assessment, tail clips for genotyping will be taken from the all the pups used in the behavioural assessments. As previously stated, we are not expecting pup genotype to influence the behaviour of the care giver due to a combination of pup age at testing and KO targeted explicitly (to the best of our knowledge) to neurons involved in parental care‐giving.

### 2.12 FFPE Tissue Preparation

All animals will be transcardially perfused with 10% neutral buffered formalin (NBF) before whole brains are taken. Using a brain slicing matrix (Zivic Instruments) a 3‐mm section of tissue will be taken with the POA situated in the centre of the block. 3‐mm sections will undergo standard pre‐paraffin embedding procedures and sectioned at a thickness of 10 μm with every 8th section used for H&E staining and every 9th and 10th section mounted on one slide for RNAscope.

### 2.13 RNAscope

Two‐plex RNA Scope will be performed using RNAscope Multiplex Fluorescent Reagent Kit v2 (ACD Bio‐techne) on FFPE brain sections following manufacturer's protocol with the ‘standard’ pre‐treatment guidance. Slides will undergo deparaffinization and H_2_0_2_ incubation before being added to boiling RNAscope Target Retrieval for 15 min, then a 30 min incubation with Protease Plus at 40°C in the HybEZ oven. Probes were incubated for 2 h at 40°C in the HybEZ oven. Each slide will contain two adjacent sections. One section will receive the probe mix, whereas the other section will receive just the probe dilutant to act as a negative control. Tissue will either receive a Gal/*Asb4* probe mix for KO validation, or a Gal/Fos probe mix for the pup‐exposure experiment. HRP‐C2 will correspond to the Gal probe (ACD 400961‐C2, Mm‐Gal‐C2) paired with the fluorophore TSA Vivid 570. HRP‐C3 will correspond to the *Asb4* probe (ACD 435091‐C3, Mm‐Asb4‐C3) and will be visualized with TSA Vivid 650 fluorophore. All fluorophores will be added at a concentration of 1:1500. Slides will be counterstained with DAPI and mounted with Prolong Gold Antifade mounting medium.

c‐Fos production in neurones will be quantified using RNAScope. Mice from each genotype will be permanently cohoused with a WT non‐sibling mate to produce litters. When pups are P1–P2, test mice will be habituated by placing the cage in the test room for 30 min with mice and pups in the cage. For each test mouse, pups and mates will be removed from the home cage and housed in a new clean cage. Pup‐exposed test animals will be left for 1‐h isolation the home cage and exposed to no stimuli in the form of pups, other mice or the experimenter. After which, three pups will be returned to the home cage with the test animal and placed in the opposite side of the cage from the undisturbed home nest. Test animals will then undergo 30‐min pup‐exposure which will begin when the test animal first investigates the pups. Once the 30 min are up, pups will be removed, and pup‐exposed test mice will be transported for perfusion and tissue harvest. Non‐pup exposed animals will function as controls and will be habituated in the same manner: isolated for 1 h in the test room and transported directly from the home cage for tissue harvest.

### 2.14 RNAscope Image Acquisition and Analysis

Whole brain slides will be imaged within 1 week of probing using Zeiss AxioScan Z1 at 20× magnification with the same light intensity settings used for each scan. Images will be analysed with Zen Blue 3.5 software. Images will be pre‐processed using a 50‐pixel radius rolling ball background correction followed by Gauss smoothing. The MPOA will be manually defined as the region of interest (ROI) for analysis. Nuclei within the ROI were localized using DAPI signal intensity and a 25 μm border will be placed around each nucleus as an estimated cytoplasm. For every cell (nucleus plus cytoplasm) the intensity of pixels within a cell will be quantified and the maximum intensity value and average intensity values for each of the channels will be calculated. The maximum intensity value for each cell in the no‐probe control will be used to calculate a threshold value for each channel, by taking the average maximum intensity value for cells in the no‐probe control plus three standard deviations. For probed genes, fluorescent pixels within the 25 μm border (cytoplasm) will be quantified as molecule counts from individual signals (punctate staining). If probes produce a diffuse or coalescent staining, signals will be resolved into molecule counts using the guidance from ACD Bio‐techne (SOP 45‐006).

*Asb4* molecule counts will be analysed in two ways. Firstly, the number of c‐Fos molecules per Gal^+^ neuron will be normalized to counts per 1000 pre‐optic area (POA) cells to account for variability in sections and POA position per animal. Secondly, as advised by ACD Bio‐techne, a semi‐quantitative metric (H‐Score) will be used to compare groups. The H‐score accounts for heterogeneous gene expression among the same cell type and will be derived from the percentage of cells: with 0 *Asb4* molecules multiplied by zero, 1–3 molecules multiplied by one, 4–9 molecules multiplied by two, 10–15 molecules multiplied by three and 16+ molecules multiplied by four. The larger the H score, the more *Asb4* molecules are present. Statistical analysis of c‐Fos will be carried out using three‐way ANOVA, with genotype, exposure and sex as variables, followed by post hoc Bonferroni‐corrected pairwise *t*‐tests.

### 2.15 Sample Size Rationale

For behaviour studies, we propose an *N* of 32 animals per genotype be used. We plan to examine a variety of parenting behaviours in our study, producing data that will require both parametric and non‐parametric models. As such, several power analyses were calculated for each proposed statistical test (summarized in Figures 1 and 2). For all calculations, an alpha of 0.05 and power (1 − β) of 0.9 were specified. Where appropriate effect size was specified as large. Larger effect sizes were chosen for power calculations based on our previous study (Higgs et al. 2023), which found robust effects for most parameters analysed. ANOVA calculations were carried out using G*Power v3.1.9.7. Cox proportional hazards model, which was done using online calculator at https://www.sample‐size.net/. For the Kruskal–Wallis test, power was estimated via Monte Carlo simulation in R (10,000 iterations). Four genotype groups were simulated: three controls (mean = 0) and one KO group shifted by *δ* ≈0.924 (Cohen's *f* = 0.4, large effect). A Kruskal–Wallis test was applied to each dataset, and power was estimated as the proportion of *p* < 0.05. Summary table of power calculations can be found in Tables 2 and 3.

**TABLE 2:** Summary table of power analysis calculations for behavioural studies on mothers and fathers.

**One‐way ANOVA** ^a^	
Effect size (*f*)	0.4
α	0.05
(1 − β)	0.90
Number of groups	4
Total sample size	96
**Sample size per genotype**	**24**
**Kruskal–Wallis test** ^a^	
Gamma distribution (for positively skewed continuous variable, e.g., time‐to‐retrieve)	32
Negative binomial distribution (for counts data, e.g., number of pups retrieved)	16
Beta distribution (for proportions, e.g., preference score)	32
**Sample size per genotype**	**32**
**Power calculations for Cox proportional hazards analysis** ^b^	
α (two‐tailed)	0.05
(1 − β)	0.1
q1	0.5
q0	0.5
RH	0.24
**Number of events, given relative hazards = 21**	
q1	0.5
Q0	0.5
RH	0.24
Total number of events	21
BER_0_	0.78
ST_0_	0.889
CR	0.2
FU	1
Sample size, given number of events = 64 animals in total	
**Sample size per genotype**	**32**

^a^ Calculated by G*Power v3.1.9.7.

^b^ Calculated by https://www.sample‐size.net/.

**TABLE 3:** Summary table of power analysis calculations for behavioural studies on virgin females.

**Two‐way mixed ANOVA** ^a^	
Effect size (*f*)	0.4
α	0.05
(1 − β)	0.90
Number of groups	8
Number of measurements	2
Corr among rep measures	0.5
Nonsphericity correction	1
Total sample size	40
**Sample size per genotype**	**10**
**Friedman test** ^a^	
Effect size (*f*)	0.4
α	0.05
β	0.90
Number of groups	8
Number of measurements	2
Corr among rep measures	0.5
Total sample size	**96**
**Sample size per genotype**	**24**

^a^ Calculated by G*Power v3.1.9.7.

For the c‐Fos analysis, we propose the use of 24 animals per genotype (six animals per group; i.e., six male no‐pup exposure, six male pup exposure, six female no‐pup exposure, six female pup exposure). An alpha of 0.05 and power (1 − β) of 0.9 and effect size large were specified. All calculations were carried out using G*Power v3.1.9.7 (Table 4).

**TABLE 4:** Summary table of power analysis calculations for c‐FOS.

**Three‐way ANOVA** ^a^	
Effect size (f)	0.4
α	0.05
β	0.90
Numerator df	3
Number of groups	16
Total sample size	94
**Sample size per genotype**	**24**

^a^ Calculated by G*Power v3.1.9.7.

Due to the use of neuron‐specific KO animals proposed in this study, we predict *Asb4* KO to have little to no effect on female mice to birth viable litters of at least three pups. We expect NO effects on mating as the mothers will be *Asb4* ^flox/+^ and so *Asb4* gene expression should be normal.

### 2.16 Exclusion Criteria

Dams who have a very small litter (< 4 pups) will be excluded. Animals that fail to habituate (defined excessive freezing or as spending more than 75% of habituation time immobile) and/or demonstrate high levels of anxiety (such as repeated self‐grooming, defecation or escape attempts during habituation time, pre‐experiment) will also be excluded. Experiment will be stopped prematurely should the adult mice start showing aggression towards the pup(s). Excluded animals will be replaced. Pairings shall be staggered to limit excess animals, make workload manageable and facilitate the replacement of excluded animals. All criteria will be applied blindly to group identity. All exclusions will be listed in supplementary data.

### 2.17 Variables

Offspring from *Asb4* ^flox/+^ females crossed with Syn1‐cre males will produce the animals used in behavioural studies. These offspring will be one of four genetic groups, summarized in Table 5. Control mice (WT) are animals that inherited *Asb4* WT allele from both dam and sire. These will function as a basal control for behaviour experiments. *Asb4* ^flox/+^ mice are animals that inherited *Asb4* ^flox^ allele from dam and WT allele from sire. As these animals do not contain the Cre recombinase these animals will be functionally WT. Syn1‐Cre mice are animals that inherited WT *Asb4* allele from dam and Syn1‐Cre transgene from sire. As these animals do not contain the *Asb4* gene with LoxP cites, they will be functionally WT. The final group will be *Asb4* ^flox/+^/Syn1‐Cre. These mice are animals that inherited *Asb4* ^flox^ allele from dam and Syn1‐Cre allele from sire. These mice will make up the experimental group containing maternal *Asb4* KO. All animals will be on a C57BL/6J background. Although tested in the KO validation stage, we will not include animals with a paternally inherited *Asb4* ^flox^ allele (e.g., *Asb4* ^*+/flox*^/Syn1‐Cre mice) as we feel these are not an essential control for the behaviour and would require separate breeding, resulting in excess unused animals.

**TABLE 5:** Establishing mouse groups for behavioural and neural analyses.

Dam allele	Sire allele	Genotype	Description
WT	WT	WT	True wild‐types; basal control
*Asb4* ^flox/+^	WT	*Asb4* ^flox/+^	Functionally WT, control for flox
WT	Syn1‐Cre	Syn1‐Cre	Functionally WT, control for cre
*Asb4* ^flox/+^	Syn1‐Cre	*Asb4* ^flox/+^ /Syn1‐Cre	Experimental animals

Dams display very robust, well‐documented behaviour; as such, any deviation is apparent. Furthermore, we are targeting a single gene KO targeted to neurons. As such, a suitable positive control does not exist to the best of our current knowledge.

Behaviours such as grooming, crouching and (females only) nursing demonstrate a desire to interact with young and increase the likelihood of optimal development and survival in offspring. Therefore, additional qualitative behaviour characteristics will be assessed as exploratory outcomes to refine the evaluation of *Asb4* effect on parenting behaviour (Table 6). Non‐parental behaviours self‐grooming and climbing cage/digging will also be assessed.

**TABLE 6:** Qualitative parental behaviour characteristics.

Behaviour	Definition	Category
Behaviours monitored during the retrieval and nest building assessment		
Licking/grooming	Parent touches pup's body with their tongue/handles pup's body with their forepaws or nose	Parental caregiving behaviour
Active nursing	Dam presents an upright dorsal arch posture with the depressed head posture over the pups. Pups are attached to the nipples	Parental caregiving behaviour
Passive nursing	Dam lies immobile on pups. Eyes can be open or closed	Parental caregiving behaviour
Self‐grooming	Licking, brushing or scratching, fur or paws using their tongue or paws. Inside or outside the nest area	Self‐maintenance
Climbing/digging	Parent climbs with all four paws attached to the cage/Dam uses forepaws to scratch sawdust away from their body	Neglecting/stressed behaviour
Behaviours monitored during the three chambers pup preference assessment		
Zone entry counts	The number of entries into pup zone vs. novel object zone to capture exploration behaviour.	Parental caregiving behaviour
Latency to first enter pup zone	Measure motivation to seek pups.	Parental caregiving behaviour
Time spent in each chamber	Assess overall chamber preference beyond the 15 cm zone.	Parental caregiving behaviour
Transition patterns	Analyse sequences of movement between chambers, which might reveal approach/avoidance behaviours.	Parental caregiving behaviour
Velocity variability	Examine changes in velocity over time as a sign of anxiety or arousal.	Neglecting or stressed behaviour

### 2.18 Behavioural Statistical Models

Effect sizes will be reported for all significant comparisons. Hypothesis will be supported if *p* > 0.05 and minimum effect size is reached. The minimum effect sizes required are summarized in Table 7.

**TABLE 7:** Minimum effect size required to support hypothesis(es).

Analysis/model type	Minimum effect size
GLM (Gamma, log‐normal or negative binomial)	Standardized *β* ≥ 0.3
Cox proportional hazards model	HR ≤ 0.70 or ≥ 1.43 (≈ Cohen's *d* of 0.3)
Cumulative link model/ordinal logistic regression	Odds ratio ≤ 0.5 or ≥ 2.0 or standardized *β* ≥ 0.3
GLMM (for repeated measures or hierarchical data)	*β* ≥ 0.3 or *R* ^2^ ≥ 0.06
Zero‐inflated GLM/GLMM	Standardized *β* ≥ 0.3
Beta regression (proportion/preference ratios)	Standardized *β* ≥ 0.3 or Δ ≥ 0.1 (raw units)
Cumulative link mixed models (ordinal repeated measures)	Standardized *β* ≥ 0.3
Equivalence testing (TOST)	Within ±0.3
Spearman's rank correlation (exploratory)	*r*_s_ ≥ 0.3
Principal component analysis/factor analysis	Component loading ≥ 0.3

#### 2.18.1 Pup Retrieval Assessment

Latency to retrieve the first pup and total time to retrieve all pups will be analysed using generalized linear models (GLMs) with a Gamma distribution and log link, which appropriately model positively skewed duration data. If more than 25% of animals fail to retrieve all pups (right‐censored data), retrieval time will instead be analysed using a Cox proportional hazards model. Post hoc pairwise comparisons will be adjusted using the Holm–Bonferroni correction. Effect sizes (e.g., model‐estimated differences with 90% CIs) will be reported.

#### 2.18.2 Nest Quality

Nest quality scores (ordinal, 1–5) will be analysed using cumulative link models (ordinal logistic regression), which respect the ordered but non‐continuous nature of the data. Genotype will be included as a fixed effect, and day (if repeated) will be included as a random effect. Model‐based contrasts will be adjusted for multiple comparisons using the Holm–Bonferroni.

#### 2.18.3 Qualitative Parental Behaviours

The distribution of behavioural categories across genotypes will be compared using GLMs with Poisson or negative binomial distributions, depending on dispersion characteristics. Predictors will include genotype, retrieval latency and number of pups retrieved. Genotype × behaviour type interactions will be tested to determine whether specific genotypes exhibit distinct behavioural profiles (e.g., altered caregiving vs. stress behaviours).

If zero inflation is present, zero‐inflated GLMs will be fitted. Model fit and residual diagnostics will guide final model selection.

#### 2.18.4 Three‐Chamber Pup Preference Test

Measures such as time spent in each chamber, interaction duration, preference ratios and locomotor indices (velocity, distance) will be analysed using GLMs or generalized linear mixed models (GLMMs) depending on design complexity. Proportion data (e.g., pup preference ratios) will be analysed using Beta regression. Latency or duration measures will use Gamma or log‐normal GLMs. Repeated measures (e.g., multiple trials or conditions) will include random intercepts for subject ID. To reduce dimensionality and identify behavioural patterns, correlated variables (e.g., time in zones, preference scores, chamber transitions) will be summarized via principal component analysis (PCA) or similar dimensionality reduction. Extracted components will then be analysed using GLMs or GLMMs to test genotype effects.

#### 2.18.5 Virgin Females—Repeated Measures Analysis

Repeated measures data (e.g., Day P3 vs. P4) will be analysed using GLMMs with the appropriate distributional family. Gamma for time‐based measures (retrieval latency, total retrieval time). Beta for proportional outcomes (preference scores). Gaussian for approximately symmetric measures. Fixed effects will include genotype, day and their interaction, with random intercepts for subject ID. Holm–Bonferroni correction will be used for post hoc contrasts. Effect sizes (standardized mean differences or model‐based estimates) with 90% confidence intervals (CIs) will be reported.

#### 2.18.6 Repeated Measures for Nest Quality and Qualitative Behaviours

For repeated ordinal measures (e.g., nest quality across days), cumulative link mixed models will be applied. For repeated count data (e.g., grooming behaviours), GLMMs with Poisson or negative binomial distributions will be used. When only a single time point is available, simple GLMs with the appropriate family will be applied.

#### 2.18.7 Test of Equivalence

Following non‐significant null hypothesis testing results, we will assess whether group differences were negligible using equivalence testing alongside estimation‐based approaches. We will use two complementary methods:

Two one‐sided tests (TOST) procedure. Equivalence will be evaluated using the TOST procedure with equivalence bounds defined a priori based on the *smallest effect size of interest* (SESOI). For behavioural outcomes, bounds corresponded to standardized effect sizes of ±0.3 (small‐to‐moderate effect) or raw units where relevant (e.g., ±10 s for retrieval latency).

CI approach. In parallel, we reported 90% CIs for all group differences. Effects were considered equivalent if the entire CI fell within the equivalence bounds.

#### 2.18.8 Exploratory Analyses

Exploratory analyses will focus on visual data exploration rather than inferential testing.

Relationships among behavioural domains (e.g., retrieval latency vs. preference score, nest quality vs. latency, motility vs. pup‐zone time) will be examined using scatterplots with nonlinear smoothers (e.g., LOESS or GAM curves) to visualize potential trends of any shape. Spearman rank correlations or nonparametric smoothing visualizations will be used descriptively.

To identify latent behavioural dimensions, PCA or factor analysis will be applied to correlated measures. These data‐driven components will guide hypothesis generation for future confirmatory studies.

#### 2.18.9 c‐Fos Expression Analysis

Normalized c‐Fos expression data will be analysed using a GLM or GLMM framework, depending on data structure, with genotype (WT vs. KO), exposure (pup vs. no pup) and sex (male vs. female) as fixed effects. Because c‐Fos counts are non‐negative and often right‐skewed, a Gamma or negative binomial distribution with a log link will be used. Model fit and residuals will be checked visually (e.g., via QQ plots, fitted vs. residual plots) rather than by formal assumption tests. Significant main effects and interactions will be followed by Holm–Bonferroni–corrected post hoc comparisons based on model‐estimated marginal means. Effect sizes will be reported as standardized mean differences (Cohen's *d*) or model‐estimated contrasts with 90% CIs.

For non‐significant results, equivalence testing (TOST) with SESOI‐defined bounds (e.g., ±0.3 standardized units) will be used to assess practical equivalence between groups. The hypothesis (H4) will be supported if KO mice show a significantly altered normalized c‐Fos count following pup exposure compared with WT mice.

## 3 Timeline

We propose a 12‐month timeline to complete the study and prepare manuscript for publication.

- Establish mouse knockout line in Cardiff (3 months)
- Validate mouse knockout line (1 month)
- Set up mouse crosses (2 months)
- Complete behavioural studies and molecular work (3 months)
- Analysis results (1 month)
- Prepare manuscript for publication (2 month)

## Author Contributions

**Rachel‐Ann Jones:** data curation (lead), formal analysis (lead), investigation (lead), methodology (lead), project administration (lead), validation (lead), visualization (lead), writing – original draft (lead), writing – review and editing (equal). **Matthew J. Higgs:** conceptualization (supporting), methodology (supporting). **Rosalind M. John:** conceptualization (equal), funding acquisition (supporting), writing – review and editing (supporting). **Anthony R. Isles:** conceptualization (lead), funding acquisition (lead), methodology (supporting), project administration (supporting), supervision (lead), writing – review and editing (equal).

## Funding

This work was supported by the Leverhulme Trust under Grant No. RPG‐2021‐041 and Wellcome (220090/Z/20/Z).

## Ethics Statement

All procedures involving animals will be conducted in accordance with local ethical guidelines and under United Kingdom Home Office project licence PP1850831 (Anthony R. Isles). The project has full funding provided by Leverhulme Trust (RPG‐2021‐041).

## Conflicts of Interest

The authors declare no conflicts of interest.

## Data Availability

All raw and analysed data files will be made available on our OSF repository (https://osf.io/zxqch/).
